# The Metabolic Syndrome and Biochemical Recurrence following Radical Prostatectomy

**DOI:** 10.1155/2011/245642

**Published:** 2011-09-25

**Authors:** Jennifer M. Post, Jennifer L. Beebe-Dimmer, Hal Morgenstern, Christine Neslund-Dudas, Cathryn H. Bock, Nora Nock, Andrew Rundle, Michelle Jankowski, Benjamin A. Rybicki

**Affiliations:** ^1^Karmanos Cancer Institute, Wayne State University, Detroit, MI 48201, USA; ^2^Department of Oncology, Wayne State University, Detroit, MI 48201, USA; ^3^Departments of Epidemiology and Environmental Health Sciences and Comprehensive Cancer Center, University of Michigan, Ann Arbor, MI 48103, USA; ^4^Department of Biostatistics and Research Epidemiology, Henry Ford Hospital, Detroit, MI 48310, USA; ^5^Department of Epidemiology and Biostatistics, Case Western Reserve University, Cleveland, OH 44109, USA; ^6^Departments of Epidemiology, Columbia University Mailman School of Public Health, New York, NY 10032, USA

## Abstract

Metabolic syndrome refers to a set of conditions that increases the risk of cardiovascular disease and has been associated with an increased risk of prostate cancer, particularly among African American men. This study aimed to estimate the association of metabolic syndrome with biochemical recurrence (BCR) in a racially diverse population. Among 383 radical prostatectomy patients, 67 patients had documented biochemical recurrence. Hypertension was significantly, positively associated with the rate of BCR (hazard ratio (HR) = 2.1; 95%  CI = 1.1, 3.8). There were distinct racial differences in the prevalence of individual metabolic syndrome components; however, the observed associations with BCR did not differ appreciably by race. We conclude that hypertension may contribute to a poorer prognosis in surgically treated prostate cancer patients. Our findings suggest that targeting components of the metabolic syndrome which are potentially modifiable through lifestyle interventions may be a viable strategy to reduce risk of BCR in prostate cancer.

## 1. Introduction

Prostate cancer is the most common invasive cancer diagnosed in men and the second leading cause of cancer death [1]. Of the men who undergo radical prostatectomy for localized prostate cancer, between 17% and 53% will experience biochemical recurrence (BCR) in the ten years following surgery [2, 3]. Traditional predictors of recurrence following radical prostatectomy include preoperative prostate-specific antigen (PSA) levels, tumor stage, Gleason's score, and surgical margin status [3, 4]. While these predictors are often used for determining BCR-free survival probabilities following radical prostatectomy [4], they are nonmodifiable characteristics of disease and as such do not provide patients with options to positively influence their disease course. Given the high level of motivation of most patients in the early postsurgery follow-up period, modifiable targets for intervention that can increase or permanently delay the time to BCR would be beneficial [5]. 

Metabolic syndrome, which is a risk factor for cardiovascular disease, refers to a clustering of conditions that include hypertension, diabetes, abdominal obesity, hypertriglyceridemia, and low high-density lipoprotein (HDL) cholesterol, with insulin resistance as the underlying hallmark feature [6]. The metabolic syndrome profile differs depending upon race, with Caucasians disproportionately affected with dyslipidemia and African Americans more likely to be diagnosed with hypertension and diabetes [7, 8]. Several studies indicate that the metabolic syndrome is associated with an increased risk of prostate cancer [9–12]. Recent findings from our own group suggest in fact that race modifies the association; metabolic syndrome was positively associated with prostate cancer risk among African American men, but not among Caucasian men [13]. 

Metabolic syndrome has an appeal as a predictor of BCR as its components can be treated and thereby provide clinicians with a strategy for tertiary prevention. To our knowledge, the association between metabolic syndrome and prostate cancer recurrence has never been systematically investigated. Therefore, the aims of this study were to estimate the effects of metabolic syndrome and its individual components on prostate cancer BCR and to determine if racial differences exist with regard to the associations of interest.

## 2. Materials and Methods

### 2.1. Study Population and Data Collection

The data for this investigation were collected as part of a prostate cancer case-control study conducted at the Henry Ford Health System (HFHS) in Detroit, Michigan, USA. HFHS provides care to a racially diverse population in the Detroit Metropolitan area [14]. Eligibility criteria for participation in the current study included (1) ≤75 years of age at time of diagnosis, (2) use of HFHS for the patient's primary medical care in the 5 years prior to diagnosis, (3) residence within the study area at time of recruitment, (4) no serious medical problems that would prohibit participation, and (5) radical prostatectomy as the patient's primary treatment. Cases were diagnosed with primary adenocarcinoma of the prostate between January 1, 1999 and December 31, 2004. The diagnosis was histopathologically confirmed by the HFHS Department of Pathology. Three hundred-ninety-six (396) prostate cancer cases were considered eligible for the current investigation based on the aforementioned criteria, with African American men comprising approximately 40% of the study population. All participants completed an interviewer-administered questionnaire. The questionnaire included information on sociodemographic characteristics, family history of prostate cancer, health behaviors including smoking history and physical activity, occupation, diet, height and weight. Data extracted from medical records included hypertension, diabetes and lipid profiles, PSA screening history, pretreatment PSA levels, clinical and pathological TNM stage, and biopsy and surgical Gleason's scores. Informed consent was gathered from all participants, and the HFHS Institutional Review Board approved all protocols.

### 2.2. Metabolic Syndrome Definition

Metabolic syndrome was defined using criteria established by the National Cholesterol Education Program Expert Panel on the Detection, Evaluation and Treatment of High Blood Cholesterol in Adults (Adult Treatment Panel III (ATP)) [15]. The ATP III definition requires any three of the following five components: (1) hypertension (≥130/85 mmHg), (2) high fasting blood glucose (≥110 mg/dL), (3) abdominal obesity (waist circumference >102 cm in men), (4) low high-density lipoprotein (HDL) cholesterol (<40 mg/dL), and (5) hypertriglyceridemia (≥150 mg/dL). In order to accommodate the available study data, specific ATP III criteria were modified. A body mass index (BMI) of greater than 30 kg/m^2^ was used as the criterion for abdominal obesity as measures of waist circumference were unavailable. BMI was calculated using self-reported height and weight at time of enrollment. A history of hypertension and/or diabetes prior to their prostate cancer diagnosis was abstracted from the medical record and recorded as present or not present.

### 2.3. Biochemical Recurrence Definition

Biochemical recurrence (BCR) was defined as two consecutive rising detectable PSA concentrations of >0.2 ng/mL [16]. Our criteria for determining the start of follow-up for identifying men at risk of BCR required that PSA levels reach a nadir ≤0.2 ng/mL after surgery. PSA levels are typically expected to drop to near undetectable levels within the four weeks following radical prostatectomy [17]. To account for the variation in the timing of PSA testing, only those subjects who reached ≤0.2 ng/mL within 6 weeks were included in this analysis. Thirteen subjects were excluded either because they did not reach a PSA nadir within 6 weeks, suggesting some residual disease or lack of PSA follow-up data, making it difficult to determine when nadir was established.

### 2.4. Statistical Methods

All statistical analyses were performed using Statistical Analysis Systems software, version 9.2 (Cary, NC, USA). Study population's demographic and clinical characteristics were described with appropriate frequency measures. Patient characteristics included age at the time of diagnosis, race, and smoking history. Clinical characteristics included preoperative PSA level, clinical stage (local, regional, distant), tumor grade, and surgical margin status. The distribution of metabolic syndrome components were examined in the total population and stratified by race. Differences in the prevalence of metabolic syndrome components between the races were evaluated with chi-square tests. 

Crude and adjusted hazard ratios were estimated using Cox regression. Time to recurrence was modeled as a function of (1) each individual component adjusted for all other components and (2) metabolic syndrome (any 3 of 5 features). Multiple models were fit, adjusting for different combinations of patient and clinical characteristics treated as potential confounders. Age at diagnosis was modeled as a continuous variable. All other covariates were included in models as dichotomous variables. Categorization of these covariates was as follows: a pre-operative PSA level of >10 ng/mL was considered high; a Gleason score 7 (4 + 3) or greater was designated high grade; regional and distant stage designations (as determined from clinical TNM and pathological staging) were categorized as high stage. Models were further stratified by race and compared to the results for the total sample.

In addition to the standardized ATP III metabolic syndrome definition examined, previous work has looked at the metabolic syndrome as an accumulation of cardiometabolic abnormalities [18]. With this in mind, the number of metabolic syndrome components was also evaluated as an ordinal variable with 3 levels: 0 (referent), 1-2, and 3 or more components.

## 3. Results

Patient and clinical characteristics for the 383 patients included in this analysis as well as the distribution of the metabolic syndrome and its components are described in Tables 1 and 2. The mean age at time of diagnosis was approximately 61 years, and as stated earlier, over 40% of the patient population was African American. Approximately 27% of patients were considered to have high-grade disease based upon their Gleason's score with 16% of patients diagnosed with regionally advanced to distant stage disease. 

Hypertension was the most commonly observed metabolic syndrome component with 56% of subjects categorized as hypertensive. There were appreciable differences in the prevalence of each component between Caucasian and African American patients (Table 2). African Americans had a higher prevalence of hypertension, obesity, and diabetes while Caucasians had a higher prevalence of low HDL and elevated triglycerides. However, there was no difference in the prevalence of metabolic syndrome overall by race.

Median follow-up time for patients in the study was 49 months (range 1 to 97 months) with 67 documented recurrences (17.5%) during the follow-up period. There was no difference between Caucasians (17.7%) and African Americans (17.3%) in the proportion of patients who recurred. The adjusted hazard ratios (HR) of BCR by metabolic syndrome component are presented in Table 3. Hypertension was associated with BCR after adjustment for patient and clinical characteristics and the other metabolic syndrome features (HR = 2.1 (95%  CI = 1.1–3.8). Low HDL level was inversely associated with the rate of BCR (Model 4: HR = 0.48; 95%  CI = 0.24–1.0). Diabetes, obesity, and high triglycerides were not associated with BCR among all patients. 

Adjusted hazard ratios for BCR are presented in Table 3 for two composite measures of metabolic syndrome. Treating the syndrome as a dichotomous measure (≥3 components versus <3 components), the estimated hazard ratio, adjusting for age, race, clinical characteristics, and smoking (Model 3), was 1.5 (95%  CI = 0.90–2.6). Treating metabolic syndrome as an ordinal variable did not reveal any trend of increasing risk of BCR with increasing number of components.

When we stratified by race, we did not find any significant differences in the association between hypertension and BCR (Caucasian: HR = 1.9; 95%  CI = 0.90–3.9 and African American: HR = 2.1; 95%  CI = 0.70–6.3; *P* interaction = 0.91). Race-stratified estimates of the association between metabolic syndrome (≥3 components) and BCR suggest a stronger association among African Americans (HR = 1.6; 95%  CI = 0.69–3.8) than Caucasians (HR = 1.2; 95%  CI = 0.56–2.5). However, our sample size was inadequate to determine the significance of the differences in the association (*P* interaction = 0.41).

## 4. Discussion

Ours is the first study to examine the association between the metabolic syndrome and BCR. We observed a 50% increase in the rate of BCR among patients classified as having metabolic syndrome. That finding was primarily influenced by the apparent effect of one metabolic syndrome component—hypertension, which was associated with an approximate 2-fold increase in the rate of BCR for both white and African American men. 

Approximately 18% of men had evidence for BCR based upon our definition. We found no appreciable difference in the BCR rate between African American and white men. This is similar to findings from two studies that showed race does not appear to be a risk factor for BCR [19, 20]. 

The positive association between hypertension and BCR was the only consistent observation among all patients across all models. Hypertension has been reported to be associated with prostate cancer risk [10] and more aggressive tumor characteristics [21]. Furthermore, antihypertensive medication is associated with a reduced risk of prostate cancer although this relation has not been examined with recurrence [22, 23]. Hypertension may promote recurrence through pathways linked to oxidative stress, whereby reactive oxygen species and low bioavailability of antioxidants have been hypothesized to promote prostate cancer cell growth [24]. 

Limitations of this analysis are important to consider for interpretation of the results. The study was designed to estimate the effects of genetic and environment factors on the risk of prostate cancer in a case control setting. Thus, our analysis of biochemical recurrence in a relatively small subsample of cases has limited statistical power to detect associations with the metabolic syndrome and its components, especially when adjusting for several potential confounders and stratified by patients' race. The potential presence of detection bias cannot be ruled out as men who have hypertension may be more likely to see a physician and, therefore, more likely to have PSA follow-up testing. To address this issue, we examined the frequency of PSA tests in the two-year period after surgery and found the mean number of tests between patients with and without hypertension was nearly identical (4.36 (SD = 1.81) versus 4.37 (SD = 1.93); pooled *t*-test *P* = 0.94). Analyses comparing postsurgery testing between men with and without metabolic syndrome (≥3 components) produced similar results (*P* = 0.49). Moreover, a previous investigation of the PSA screening behavior of these patients did not suggest any difference in the frequency of PSA testing prior to diagnosis [13]. 

Lipid profiles were incomplete for 45 subjects, and this missing lipid data would likely result in an underestimation of those subjects classified as having metabolic syndrome. Additionally, BMI was calculated based on self-reported height and weight. Classifications of abdominal obesity estimated by BMI could be inaccurate. Abdominal obesity is less common in African American men than other racial groups [7]. Furthermore, BMI is considered a suboptimal measure for abdominal obesity, particularly in African American men because visceral fat is most closely linked with altered lipid concentrations and insulin resistance [25].

Timing of PSA followup is another limitation of this analysis. As an observational investigation the PSA followup was done at the discretion of the treating physician and limited by subject compliance; the cases were tested for recurrence at irregular intervals. Similarly, cases with limited PSA follow-up data were excluded from our analyses. It is possible, therefore, that undetected BCR events might have biased the results if PSA followup is more or less likely to occur based upon the existence of the metabolic syndrome conditions. It is important to note, however, that there were few exclusions based upon limited data, and cases with lengthy intervals (>12 months) between PSA tests were a small proportion of subjects included.

Among the strengths of this investigation is the reliability of clinical data for determination of metabolic syndrome and BCR. Hypertension, diabetes, and lipid profiles were abstracted directly from the medical record. Additionally, PSA results were available for subjects for a median of 4 years after diagnosis. Results from prior investigations indicate the majority of localized prostate cancers that recur after radical prostatectomy are detected soon after surgery [26]. The racially diverse study population is another major strength of the investigation as the large percentage of African American participants makes it ideal to evaluate the influence of race on BCR.

## 5. Conclusions

This investigation was the first to evaluate metabolic syndrome and its components as predictors of the biochemical recurrence of prostate cancer after radical prostatectomy in both African American and Caucasian men. Metabolic syndrome was modestly, but not significantly, associated with increased BCR, regardless of race. Of the individual metabolic syndrome components, hypertension was consistently associated with increased BCR. Further investigations of metabolic syndrome and BCR in larger populations are needed to replicate these findings; if validated, the medical management of hypertension could influence the long-term prognosis of men with prostate cancer after definitive treatment.

## Figures and Tables

**Table 1:** Frequency distribution (number and percent) of demographic and clinical characteristics among radical prostatectomy patients (*n* = 383).

Characteristic	All men		Recurrence		No recurrence	
	*N*	%	*N*	%	*N*	%
Age*	60.9	10.1	60.6	5.9	60.9	6.8
Race						
Caucasian	215	56.1	38	56.7	177	56.0
African American	168	43.9	29	43.3	139	44.0
Smoking history						
Ever	249	65.0	51	76.1	198	62.7
Never	134	35.0	16	23.9	118	37.3
Pretreatment PSA (ng/ml)						
≤10	331	86.4	50	74.6	281	88.9
>10	52	13.6	17	20.4	35	11.1
Gleason score						
≤7 (3 + 4)	280	73.1	41	61.2	239	75.6
≥7 (4 + 3)	103	26.9	26	38.8	77	24.4
Stage						
Local	321	83.8	49	73.1	272	86.1
Regional	58	15.2	15	22.4	43	13.6
Distant	4	1.0	3	4.5	1	0.3
Surgical margin status						
Positive	111	29.8	38	56.7	73	23.1
Negative	262	70.2	27	43.3	235	76.9

*Mean value with standard deviation.

**Table 2:** Frequency distribution (number and percent) of metabolic syndrome features among all participants (*n* = 383) and by race.

Features	All men (*n* = 383)		White men (*n* = 215)		AA men (*n* = 168)		
	*N*	%	*N*	%	*N*	%	*P* value*
Hypertension	216	56.4	109	50.7	107	63.7	0.01
Diabetes	56	14.6	18	8.4	38	22.6	<0.0001
Obesity	121	31.6	59	27.4	62	36.9	0.05
Low HDL cholesterol	76	22.5	52	26.7	24	16.8	0.03
High triglycerides	141	41.4	93	47.2	48	27.4	0.01
Metabolic syndrome (≥3 features)	95	24.8	49	22.8	46	27.4	0.30

*Based on Mantel-Haenszel chi-square comparing racial differences in prevalence of features.

**Table 3:** Adjusted hazard ratios (95% confidence intervals) of biochemical recurrence (BCR) by metabolic syndrome component (*n* = 383).

Feature	% of sample	Model 1	Model 2	Model 3	Model 4
Hypertension	56.4	1.9 (1.1–3.3)	1.8 (1.0–3.1)	1.7 (0.99–2.9)	2.1 (1.1–3.8)
Diabetes	14.6	1.0 (0.52–2.1)	0.99 (0.48–2.0)	1.1 (0.52–2.2)	0.99 (0.48–2.1)
Obesity	31.6	1.1 (0.63–1.8)	1.0 (0.59–1.7)	1.0 (0.60–1.8)	0.97 (0.55–1.7)
Low HDL cholesterol	22.5	0.68 (0.36–1.3)	0.57 (0.30–1.1)	0.58 (0.30–1.1)	0.48 (0.24–1.0)
High triglycerides	41.4	0.90 (0.54–1.5)	0.92 (0.54–1.6)	0.92 (0.54–1.6)	1.1 (0.64–1.7)

*Metabolic Syndrome *					
<3 features (referent)	75.2	1.0	1.0	1.0	
≥3 features	24.8	1.4 (0.83–2.4)	1.4 (0.85–2.5)	1.5 (0.90–2.6)	—

*Ordinal Model*					
0 features (referent)	23.2	1.0	1.0	1.0	
1-2 features	52.0	0.96 (0.52–1.8)	0.85 (0.45–1.6)	0.75 (0.39–1.4)	—
≥3 features	24.8	1.4 (0.70–2.7)	1.3 (0.65–2.6)	1.3 (0.63–2.5)	—

Model 1: adjusted for age and race.

Model 2: adjusted for age, race, and clinical characteristics (pre-operative PSA, Gleason's grade, tumor stage, surgical margin status).

Model 3: adjusted for age, race, clinical characteristics and smoking.

Model 4: adjusted for age, race, clinical characteristics, smoking, and other metabolic syndrome components.
